# Supplementary material for: Does the presence of general practitioners in emergency departments affect quality and safety in English NHS hospitals? A retrospective observational study
Source: BMJ Open. 2022 Feb 23;12(2):e055976. doi: 10.1136/bmjopen-2021-055976 (PMC8867306; doi:10.1136/bmjopen-2021-055976)
Supplement: Supplementary data [file bmjopen-2021-055976supp001.pdf]

Appendix A: Detailed description of outcome definitions

ID	Outcome name	Algorithm (variables used in parentheses)	Causes of missing values
1	Wait over 4 hours	= 1 if the time from admission to discharge (depdur) in an ED is greater than four hours.	depdur is missing (value of .)
2	Unplanned re-attendance within 7 days	= 1 if the patient (extract_hesid) re-attended an ED within 7 days of discharge (difference in arrivaldate) and that reattendance was not planned (aeattendcat = 1 or 3).	aeattendcat is unknown (value = 9)
3	Untreated	= 1 if the attendance is unplanned (aeattendcat =1 or 3) and the patient leaves before being treated (aeattenddisp = 12)	Aeattendcat is unknown (value 9) or aeattenddisp is unknown (value 99)
4	'Unnecessary' attendance	=1 if all of the following conditions are met:<br><br>1. Any investigations (invest_nn) reported are for one of (have the first two characters) "urin analysis" 06, "pregnancy test" "21", dental investigation" (22) "none" (24).<br><br>2. Any treatments (treat_nn) are limited to (a recorded values of) "guidance/advice only-written" (221), "guidance/advice only-verbal (222), "recording vital signs" (30), "dental treatment" (56), "prescription/medicine prepared to take away (57), "none" (99)<br><br>3. Patient not treated or any follow-up limited to primary care (aeattenddisp values of) "discharged-follow up	n/a, as it is not possible to disentangle nul from missing values.

		treatment to be provided by general practitioner (02), “discharged did not require any follow-up treatment” (03), “left department before being treated” (12)<br><br>4. Attendance is not by ambulance (aearrivalmodel doesn’t equal 1)	
5	Admission to ward	=1 if patient is admitted as an inpatient following ED attendance (aeattenddisp = 1 or 7).	aeattenddisp is unknown (value 99)
6	30-day mortality	=1 if date of death of the patient is within 30 days after ED attendance.	
7	Volume of attendances	Count of attendances per hour of day and day of week	
