# Supplementary material for: Does the presence of general practitioners in emergency departments affect quality and safety in English NHS hospitals? A retrospective observational study
Source: BMJ Open. 2022 Feb 23;12(2):e055976. doi: 10.1136/bmjopen-2021-055976 (PMC8867306; doi:10.1136/bmjopen-2021-055976)
Supplement: Author's manuscript [file bmjopen-2021-055976.draft_revisions.pdf]

BMJ Open is committed to open peer review. As part of this commitment we make the peer review history of every article we publish publicly available.

When an article is published we post the peer reviewers' comments and the authors' responses online. We also post the versions of the paper that were used during peer review. These are the versions that the peer review comments apply to.

The versions of the paper that follow are the versions that were submitted during the peer review process. They are not the versions of record or the final published versions. They should not be cited or distributed as the published version of this manuscript.

BMJ Open is an open access journal and the full, final, typeset and author-corrected version of record of the manuscript is available on our site with no access controls, subscription charges or pay-per-view fees (http://bmjopen.bmj.com).

If you have any questions on BMJ Open's open peer review process please email info.bmjopen@bmj.com

BMJ Open

Does the presence of General Practitioners in Emergency Departments affect quality and safety in English NHS Hospitals? A panel data model

Journal:	*BMJ Open*
Manuscript ID	bmjopen-2021-055976
Article Type:	Original research
Date Submitted by the Author:	02-Aug-2021

Complete List of Authors:	Gaughan, James; University of York Centre for Health Economics,<br>Liu, Dan; Centre for Health Economics, University of York<br>Gutacker, Nils; University of York, Centre for Health Economics<br>Bloor, Karen; University of York, Department of Health Sciences<br>Doran, Tim; University of York, Department of Health Sciences<br>Benger, Jonathan; University of the West of England, Faculty of Health and Applied Sciences

Keywords:	Health policy < HEALTH SERVICES ADMINISTRATION & MANAGEMENT, ACCIDENT & EMERGENCY MEDICINE, PRIMARY CARE, HEALTH ECONOMICS

SCHOLARONE™
Manuscripts

I, the Submitting Author has the right to grant and does grant on behalf of all authors of the Work (as defined in the below author licence), an exclusive licence and/or a non-exclusive licence for contributions from authors who are: i) UK Crown employees; ii) where BMJ has agreed a CC-BY licence shall apply, and/or iii) in accordance with the terms applicable for US Federal Government officers or employees acting as part of their official duties; on a worldwide, perpetual, irrevocable, royalty-free basis to BMJ Publishing Group Ltd ("BMJ") its licensees and where the relevant Journal is co-owned by BMJ to the co-owners of the Journal, to publish the Work in this journal and any other BMJ products and to exploit all rights, as set out in our licence.

The Submitting Author accepts and understands that any supply made under these terms is made by BMJ to the Submitting Author unless you are acting as an employee on behalf of your employer or a postgraduate student of an affiliated institution which is paying any applicable article publishing charge ("APC") for Open Access articles. Where the Submitting Author wishes to make the Work available on an Open Access basis (and intends to pay the relevant APC), the terms of reuse of such Open Access shall be governed by a Creative Commons licence – details of these licences and which Creative Commons licence will apply to this Work are set out in our licence referred to above.

Other than as permitted in any relevant BMJ Author's Self Archiving Policies, I confirm this Work has not been accepted for publication elsewhere, is not being considered for publication elsewhere and does not duplicate material already published. I confirm all authors consent to publication of this Work and authorise the granting of this licence.

For peer review only

Does the presence of General Practitioners in Emergency Departments affect quality and safety in English NHS Hospitals? A panel data model

James Gaughan[1], Dan Liu[1], Nils Gutacker[1], Karen Bloor[2], Tim Doran[2], Jonathan Benger[3]

[1] Centre for Health Economics, University of York, York, UK
[2] Health Sciences, University of York, York, UK
[3] Faculty of Health and Applied Sciences, University of the West of England, Bristol, UK

Corresponding Author: James Gaughan, Alcuin 'A' Block, University of York, Hslington, York, UK, YO10 5DD, telephone: 01904 321423

Key words: Health policy; Accident and Emergency Medicine; Primary Care; Health Economics

Word count: 2851

For peer review only

Abstract

Objectives
Emergency departments (EDs) in NHS hospitals in England have faced considerable increases in demand over recent years. Most hospitals have developed General Practitioner services in EDs (GPEDs) to treat non-emergency patients, aiming to relieve pressure on other staff and to improve ED efficiency and patient experience. We measured the impact of GPED services on patient flows, health outcomes and ED workload.

Methods
Retrospective observational study of clinical activity in EDs in English NHS hospitals from April 2018 to March 2019. Differences in GPED service availability across EDs and time-of-day were used to identify the causal effect of GPED, as patients attending the ED at the same hour of the day are quasi-randomly assigned to treatment or control groups based on their local ED's service availability. Outcomes measured were volume of attendances, 'unnecessary' attendances, waiting times over four hours, patients leaving without being treated, unplanned re-attendances within seven days, inpatient admissions and 30-day mortality.

Results
We found a small, statistically significant reduction in unplanned re-attendances within seven days (OR: 0.968, 95% CI: 0.948 to 0.989), equivalent to 302 fewer re-attendances per year for the average ED. The clinical impact of this was judged to be negligible. There was no detectable impact on any other outcome measure.

Conclusions
Our findings do not support the widespread use of GPs in EDs in England as currently implemented.

Strengths and limitations of this study

- Large sample size (all ED attendances to 40 English hospitals over one year)
- Time of patient arrival used to support a causal interpretation
- Wide range of performance measures analysed
- Inability to identify the healthcare professional seen by patients prevented direct comparison
- Data analysis is based on a number of assumptions

Funding: This study was funded by the National Institute of Health Research (NIHR) Health Services and Delivery Research (HS&DR) Programme, project numbers 15/145/04 and 15/145/06.

Competing Interests: The authors have no competing interests to declare.

Contributorship statement: The study was conceived and designed by JB, KB, TD and NG. JG and DL prepared the dataset. JG and NG analysed the data, with additional interpretation by all other authors. JG drafted the manuscript, which was critically reviewed and revised by all other authors. All authors approved the final submission.

Data Sharing Statement: No additional data available.

Ethics Statement: This research study was approved by:

- East Midlands - Leicester South Research Ethics Committee (Ref: 17/EM/0312)
- University of Newcastle Ethics Committee (Ref: 14348/2016)
- Health Research Authority (HRA) (IRAS: 230848 and 218038).

For peer review only

Introduction

In common with many health care systems, emergency departments (EDs) in the English NHS have faced considerable growth in demand over recent years.[1] A substantial minority of attendances are suitable for treatment by a General Practitioner (GP); Mason et al[2] estimated this proportion to be around 23% of adults and 31% of children attending the ED. Efforts to reduce unnecessary attendances to EDs, such as the introduction of walk-in primary care centres and NHS telephone and online advice services, have been shown to address unmet needs but have not changed trends in ED attendance.[3] Following the recommendations of a national review of emergency services,[4] most hospital Trusts have developed GP services in EDs (GPEDs) to treat non-urgent patients, aiming to relieve pressure on other ED staff and improve ED efficiency and patient experience.[5 6] Different models of GPs working in or alongside the ED have emerged; some are fully integrated within the ED, others ‘stream’ patients either to GPs working alongside EDs or to outside services, which may be offered on site or off site.[7]

Evidence of the effectiveness of GP services in EDs is limited to date. A 2018 Cochrane review found only four studies (one randomised trial and three non-randomised trials) that evaluated the effects of introducing GPs or emergency nurse practitioners.[8] These studies did not examine safety or cost-effectiveness and had inconsistent results, leading reviewers to comment that the evidence was insufficient to draw conclusions for practice or policy. More inclusive reviews have similarly found inadequate evidence on the effectiveness of this approach.[9 10]

As part of a mixed methods study to explore the impacts of General Practitioners working in or alongside the emergency department,[11] we analysed data from hospitals with a well-established and clearly defined GPED service during the financial year April 2018 to March 2019, using the presence of a GP service as the intervention. The study aim was to measure the impact of GPED services on patient flows (including waiting time, treatment in the ED and unplanned re-attendance) and health outcomes (including 30-day mortality).

Methods

Study design and data sources

We undertook a retrospective observational study of all clinical activity in Type 1 emergency departments (i.e. EDs that are open 24 hours a day and led by a Consultant in Emergency Medicine) in English NHS hospital trusts during the period 01 April 2018 to 31 March 2019. Complete data on availability and type of GPED service, derived from a national mapping survey,[12] were available for 40 of England’s 137 Type 1 emergency departments. The unit of analysis was an individual ED attendance.

The primary data source was the Hospital Episode Statistics Accident & Emergency (HES A&E) dataset, which provides information on patients’ socio-demographic characteristics (age, sex, and local area deprivation profile), the timing of their attendance and discharge from ED (recorded as date, hour and minute) and the discharge destination. Information on the date of death for deceased patients was provided by the Office for National Statistics.

HES A&E data do not identify the treating healthcare professional and it was therefore not possible to ascertain whether a patient was seen by a GP or another member of staff. Hospital-reported GPED opening hours were used instead to assign ED attendances to treatment and control groups based on the potential exposure to GPED services at the patient’s time of arrival to the ED. Data on opening and closing hours of GPED services for individual hospitals by day of the week were obtained via the

For peer review only - http://bmjopen.bmj.com/site/about/guidelines.xhtml

mapping survey.[12] EDs were also asked to identify their local model of GPED service, which was pre-specified to be either: i) integrated, with the GP working as part of the ED team; ii) parallel, where the GP service is separate from but located adjacent to the ED; or iii) off site, with the GP service provided at a separate site on the same hospital campus.[7] Only hospital EDs that provided complete data as part of this national survey were included in the analyses.

Outcome measures

We investigated the impact of GPED on a range of different measures of ED performance and patient outcomes (*Table 1*). All outcome measures were defined at individual patient level except volume of activity, which is measured at ED level. Outcomes could not be calculated for all attendances due to missing information recorded in the HES dataset. For example, discharge destination was not recorded for 14% of patients so that outcomes 3 ('Untreated') and 6 ('Admitted to ward') could not be calculated for these patients. Observations with missing outcome information were only excluded from the statistical analysis of the relevant outcome.

For peer review only

Table 1: Outcome measures and summary statistics for all EDs in England and those included in the analysis.

ID	Outcome	Definition	Level of measurement	Proportion of patients (%)		Missing (%) (included EDs only)
				Included EDs (n=40)	Excluded EDs (n=97)	
Patient flow and outcomes						
1	Wait over 4 hours	Time from admission to discharge was more than four hours.	Patient	15.85	19.89	0.6
2	Unplanned re-attendance	Patient made an unplanned re-attendance to the same or different ED within 7 days of discharge.	Patient	8.86	8.57	3.5
3	Untreated	Patient left ED without being treated.	Patient	2.32	2.19	14.2
4	'Unnecessary' attendance	An attendance is 'unnecessary' if **all** of the following conditions are met:<br>• No Investigations were recorded or they were limited to urinalysis, pregnancy test, or dental investigation.<br>And	Patient	11.75	10.67	0.0

		• No treatments were recorded or were limited to guidance, written advice, verbal advice, recording of vital signs, dental treatment, or prescription of medicines.<br>And<br>• Treatment or follow-up was limited to primary care or patient left before being treated.<br>And<br>• Attendance was not by ambulance.				
5	30-day mortality	Date of death of the patient is within 30 days after ED attendance.	Patient	1.57	1.70	0.0
6	Admission to ward	Patient admitted as an inpatient following ED attendance.	Patient	42.43	23.54	14.2
ED workload						
ID	Outcome	Definition	Level of measurement	Mean (SD)		Missing (%) (included EDs only)
				Included EDs (n=40)	Excluded EDs (n=97)	
7	Volume of attendances	Count of attendances per hour of day and day of week	Provider-hour-day	13.50 (9.30)	14.42 (11.71)	0.0

Statistical analysis

The statistical analysis relies on differences in GPED service availability across EDs and time-of-day to identify the causal effect of GPED on outcomes. Hospitals operate different opening hours for their GPED services and few offer 24/7 coverage. For example, a service might begin at 8am and end at 11pm in one hospital but run from 12pm to 8pm in another hospital. This created a natural experiment in which patients attending the ED at the same hour of the day are quasi-randomly assigned to treatment or control groups based on their local GPED service availability.

Logit and Poisson regression models were estimated to identify the effect of treatment status on outcomes at patient and provider level. Each outcome was analysed separately. All models controlled for hospital and hour-by-day of week fixed effects, month of year fixed effects, and, in the case of the attendance-level regressions, potential residual confounders in the form of patient age (coded in 5-

For peer review only

year age bands), sex, age-sex interactions, an indicator for arrival by ambulance, and an indicator of socio-economic deprivation. The hospital fixed effects capture time-invariant differences in performance across EDs that reflect hospital-specific factors such as management quality, building infrastructure, and the quality and availability of substitute healthcare services within the local health economy.[13] The hour-by-day fixed effects capture differences in service availability and patient acuity over the course of the day that follow a common pattern across all hospitals in England. Attendances in the last 7 or 30 days of the sample period were excluded from analysis for outcomes 7-day reattendance and 30-day mortality, respectively.

Our main analyses assumed a common effect across GPED models. Secondary analyses allowed for subgroup effects by type of GPED model.

The scope of this analysis was limited to time periods where there was variation in GPED service availability across hospitals. Patients attending EDs during hours of the day when all/none of the EDs operated a GPED service contributed to the identification of hospital fixed effects and the effect of observed confounders but did not contribute to the statistical identification of the effect of GPED services on outcomes.

Estimates are reported as odds ratios (ORs) or incidence rate ratios (IRRs) with associated 95% confidence intervals (CIs). Bonferroni-Dunn correction was applied to adjust CIs for multiple testing while ensuring an overall family-wise error rate of 5%.[14] Standard errors are clustered at ED level. All analyses were performed in Stata version 16 (StataCorp, College Station, TX, USA).

Patient and public involvement

We held meetings with a patient and public Research Advisory Group throughout the study. We discussed the study methods with this group and presented results for feedback at various stages, modifying the research and its interpretation in response to their feedback. Our final results were disseminated to the Research Advisory Group and others through a collaborative workshop. Our study was an analysis of secondary data; therefore, we did not recruit patients as research participants.

Results

GPED services

Data on GPED service opening hours and GPED model were available for 40 EDs, with a total of 4.6m attendances between April 2018 and March 2019. Most EDs had a GP present between 10am and 10pm on all days of the week (figure 1), which coincides with the time of day in which workload tends to be highest (figure 2). Some hospitals operate different GPED service opening hours during weekdays and weekends, with two hospitals offering no GPED services on weekends. One or more GPs were present in EDs in our sample for a median of 13 hours (IQR: 11 to 15) on weekdays and 13 hours (IQR: 9 to 15) on weekends.

Demographic characteristics of patients attending EDs where a GPED service was available and where GPED was not available were similar, on average, and followed a common pattern over the course of the day (*Figure 3*). Patients attending EDs where a GPED service was available during the early hours of the day were, on average, from less deprived neighbourhoods.

For peer review only

Figure 1 about here

Figure 2 about here

Figure 3 about here

Impact of GPED

The results of the main analysis are presented in table 2. There was no statistically significant effect of GPED on any of the outcome measures aside from a small, statistically significant reduction in unplanned re-attendances within seven days (OR: 0.968, 95% CI (unadjusted): 0.948 to 0.989). This effect was only detectable for integrated and parallel GPED services, and not for off-site GP services. None of these findings reach statistical significance when adjusted for multiple testing.

Table 2: Impact of GPED service availability on outcomes, overall and by GPED model

**Outcome / GPED model**	**OR / IRR**	**95% CI (unadjusted)**	**95% CI (adjusted for multiple testing)**
Wait over 4 hours			
Overall	1.012	0.927 to 1.105	0.870 to 1.154
Integrated	0.906	0.793 to 1.034	0.695 to 1.117
Parallel	0.998	0.863 to 1.132	0.784 to 1.212
Off-site	1.077	0.972 to 1.182	0.910 to 1.244
Unplanned re-attendance			
Overall	0.968	0.948 to 0.989	0.935 to 1.002
Integrated	0.931	0.891 to 0.973	0.861 to 1.001
Parallel	0.972	0.945 to 0.998	0.930 to 1.014
Off-site	0.980	0.950 to 1.009	0.933 to 1.027

For peer review only

Untreated			
Overall	0.914	0.788 to 1.061	0.698 to 1.131
Integrated	0.978	0.837 to 1.142	0.731 to 1.225
Parallel	0.927	0.730 to 1.124	0.613 to 1.241
Off-site	0.788	0.620 to 0.955	0.521 to 1.055
'Unnecessary' attendance			
Overall	1.038	0.944 to 1.142	0.881 to 1.196
Integrated	1.014	0.844 to 1.219	0.721 to 1.307
Parallel	1.093	0.999 to 1.187	0.943 to 1.243
Off-site	0.990	0.874 to 1.106	0.805 to 1.175
Admission to ward			
Overall	1.029	0.957 to 1.106	0.910 to 1.147
Integrated	1.039	1.016 to 1.185	0.916 to 1.161
Parallel	1.002	0.886 to 1.106	0.807 to 1.197
Off-site	1.047	0.957 tot 1.171	0.896 to 1.198
30-day mortality			
Overall	1.013	0.990 to 1.037	0.976 to 1.051
Integrated	1.041	0.968 to 1.119	0.925 to 1.157
Parallel	1.017	0.972 to 1.061	0.947 to 1.087

For peer review only

Off-site	1.000	0.968 to 1.032	0.948 to 1.052
Volume of attendances			
Overall	1.000	0.967 to 1.034	0.946 to 1.054
Integrated	0.983	0.914 to 1.053	0.873 to 1.094
Parallel	1.018	0.967 to 1.069	0.937 to 1.099
Off-site	0.986	0.938 to 1.034	0.910 to 1.062

For peer review only

Discussion

Statement of principal findings

Many hospitals in England have implemented GPED services over time, with the aim of treating non-urgent patients more appropriately, thereby improving efficiency and patient experience. Using attendance data for 2018/19, we found no statistically significant effect of GPED services on volume of attendances or on most performance indicators, including four-hour waits, patients leaving without being treated, or hospital admissions. We did identify a small, statistically significant reduction in unplanned re-attendances within seven days, equivalent to 302 fewer re-attendances per year for the average ED. This is less than one prevented re-attendance per day for the average ED. The finding was not statistically significant after adjusting for multiple testing, and was judged to be of negligible clinical significance.

Strengths and weaknesses

Our analysis exploited differences in the implementation of GPED services across 40 hospitals in terms of time of operation to model robustly their impact on a range of key indicators, accounting for a wide range of covariates. Using information from targeted surveys, we were also able to perform a sub-group analysis for three different GPED models, strengthening the evidence base to inform decisions about implementing GPED services.

Our analysis was, however, constrained in a number of ways. Most importantly, it was not possible to identify from available data which staff members assessed and treated individual patients, so we could not separate patients treated by GPs from those treated by other ED staff to directly compare GP services to traditional models of care. We relied primarily on measures of general ED performance, such as attendances, patient flow and waiting times. Our approach assumed GPs were present during the working hours reported in the survey, but there is potential for misclassifying patient episodes as GPED/not GPED as streaming activity and GP availability may not have corresponded exactly to official GPED start and finish times, particularly for patients arriving close to these times or undergoing extended waits. Related to this, our analysis assumes that GPED was always operational during designated hours and therefore does not take account of physician absence. There were also small differences in observed outcomes between hospitals included in our analysis and those excluded from analysis (Table 1). Finally, we were not able to assess the degree

of 'role substitution', i.e. whether GPs were an addition to normal services, or whether they replaced another member of staff.

Principal findings in context of other studies

We found that patients attending emergency departments during the hours of operation of a GPED service had similar outcomes to patients attending at other times, with the possible exception of a very small reduction in unplanned re-attendances within seven days. Previous evidence on the impact of such services is extremely limited, and no previous studies have examined interventions on the same scale. A 2018 Cochrane review found no clear evidence that deploying primary care physicians in emergency departments reduced treatment times, lengths of ED stay, safety, hospital admissions, resource use, cost-effectiveness or referrals.

Future research in this area could usefully explore the implementation of GPED services, using more qualitative methods, and include other important outcome measures (for example, those reflecting patient experience). The cost implications of these services should also be investigated further. Employing GPs in EDs has opportunity costs in terms of both alternative use of funds for urgent and emergency care (for example, employing additional nurses, physician associates or emergency physicians) and loss of GPs from primary care settings. Prior to the COVID-19 pandemic, there was a trend of increasing demand for both ED and primary care services, and general practice is an area of potential workforce shortages, with a forecasted shortfall of 7,000 GPs in the next five years.[15] This raises further questions about the appropriateness of recruiting GPs to work in hospital ED settings.

Conclusions

Our findings do not support the widespread use of GPs in EDs. Whilst we found no adverse effects on patient outcomes, neither did we find any evidence of the hypothesised benefits of placing GPs in emergency settings beyond a marginal reduction in re-attendances that was not considered clinically significant. Given that GPED services are likely to incur significant additional costs, and take GPs from primary care services which are themselves stretched, we found no evidence that they are a good use of scarce health care resources unless the model can be developed further and substantial improvements in outcomes can be achieved.

Data Sharing Statement

The deidentified patient-level data used in this study were released by the data holders (NHS Digital, Office for National Statistics) under specific data sharing agreements and only for the purpose of this study. The data sharing agreements do not permit further sharing or publication of the data. Interested parties may seek to obtain data directly from the relevant data holders. HES data are copyright 2018-2019, re-used with the permission of NHS Digital through Data Sharing Agreement NIC-84254-J2G1Q.

For peer review only

References

1. National Audit Office. Emergency admissions to hospital: managing the demand, 2013.
2. Mason S, O'Keeffe C, Jacques R, et al. Perspectives on the reasons for Emergency Department attendnaces acrss Yorkshire and the Humber - Final Report September 2017. The University of Sheffield, 2017.
3. Turner J, O'Cathain A, Knowles E, et al. Impact of the urgent care telephone service NHS 111 pilot sites: a controlled before and after study. *BMJ Open* 2013;3(11):e003451. doi: 10.1136/bmjopen-2013-003451
4. NHS England. High quality care for all, now and for future generations: Transforming urgent and emergency care services in England - Urgent and Emergency Care Review End of Phase 1 Report. Leeds, 2013.
5. NHS England. Next steps on the NHS Five Year Forward View, 2017.
6. NHS England. Transforming urgent and emergency care services in England - Safer, faster, better: good practice in delivering urgent and emergency care. London, 2016.
7. Cooper A, Edwards M, Brandling J, et al. Taxonomy of the form and function of primary care services in or alongside emergency departments: concepts paper. *Emergency Medicine Journal* 2019;36(10):625-30. doi: 10.1136/emermed-2018-208305
8. Gonçalves-Bradley D, Khangura JK, Flodgren G, et al. Primary care professionals providing non-urgent care in hospital emergency departments. *Cochrane Database Syst Rev* 2018;2(2):Cd002097. doi: 10.1002/14651858.CD002097.pub4
9. Ramlakhan S, Mason S, O'Keeffe C, et al. Primary care services located with EDs: a review of effectiveness. *Emergency Medicine Journal* 2016;33(7):495-503. doi: 10.1136/emermed-2015-204900
10. Cooper A, Davies F, Edwards M, et al. The impact of general practitioners working in or alongside emergency departments: a rapid realist review. *BMJ Open* 2019;9(4):e024501. doi: 10.1136/bmjopen-2018-024501
11. Morton KV, S. Adamson, J. Baxter, H. Bloor, K. Brandling, J. Cowlishaw, S. Doran, T. Gibson, A. Gutacker, N. Purdy, DLS. Roy, P. Salisbury, C. Scantlebury, A. Vaittinen, A. Watson, R. Benger, J. General practitioners and emergency departments (GPED) - efficient models of care: a mixed-methods study protocol. *BMJ Open* 2018
12. Brant HV, S. Morton, K. Cooper, A. Edwards, M. Price, D. Gaughan, J. Edwards A. Current provision of general practitioner services in or alongside emergency departments in England. *Emeergency Medicine Journal* 2021
13. Wooldridge JM. Econometric Analysis of Cross Section and Panel Data. Cambridge, Massachusetts: MIT Press 2002.
14. Dunn OJ. Multiple Comparisons among Means. *Journal of the American Statistical Association* 1961;56(293):52-64. doi: 10.1080/01621459.1961.10482090
15. Beech J, Bottery S, Charlesworth A, et al. Closing the gap - Key areas for action on the health and care workforce. London: The King's Fund, 2019.

For peer review only

Figure Legend

Fig 1: GPED service availability by hour of day
Fig 2: Volume of attendances by hour of day
Fig 3: Patient characteristics by hour of day (mean, 95% CI)

For peer review only

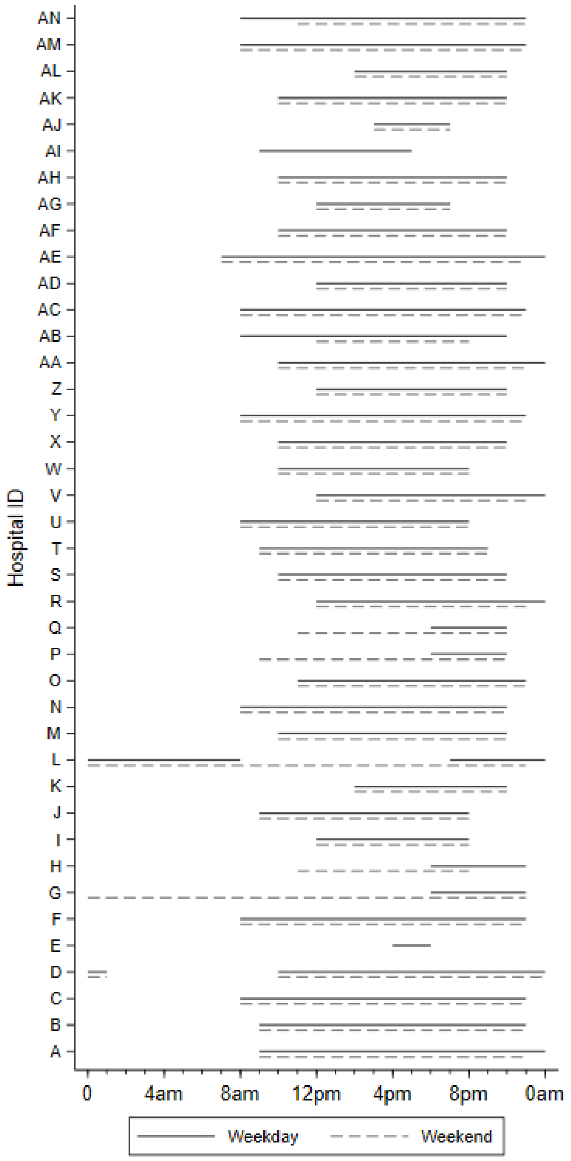

136x273mm (72 x 72 DPI)

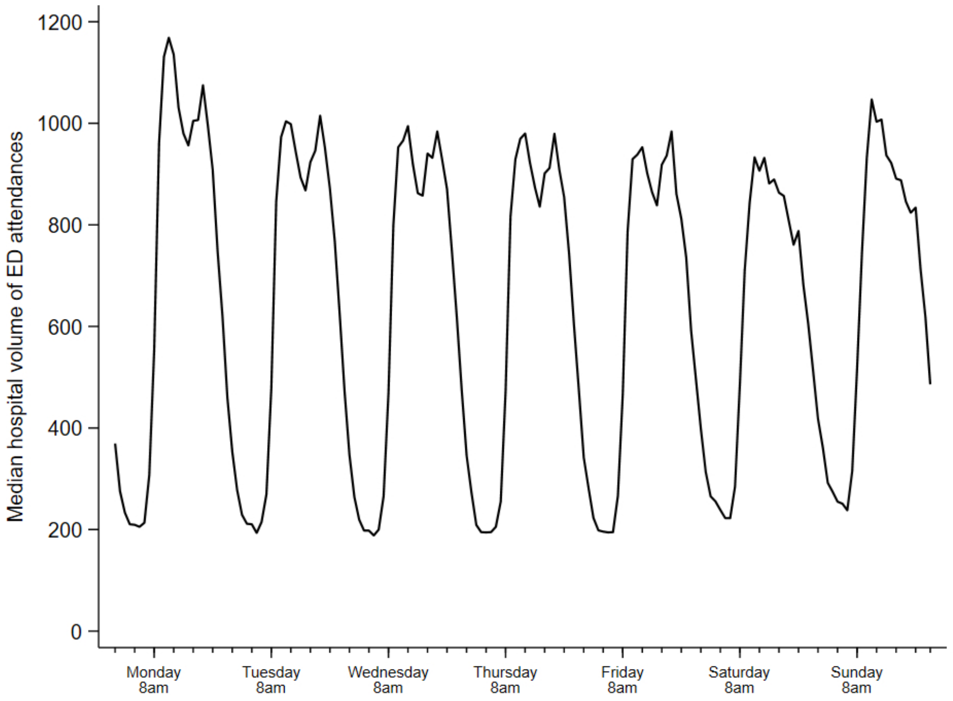

342x257mm (72 x 72 DPI)

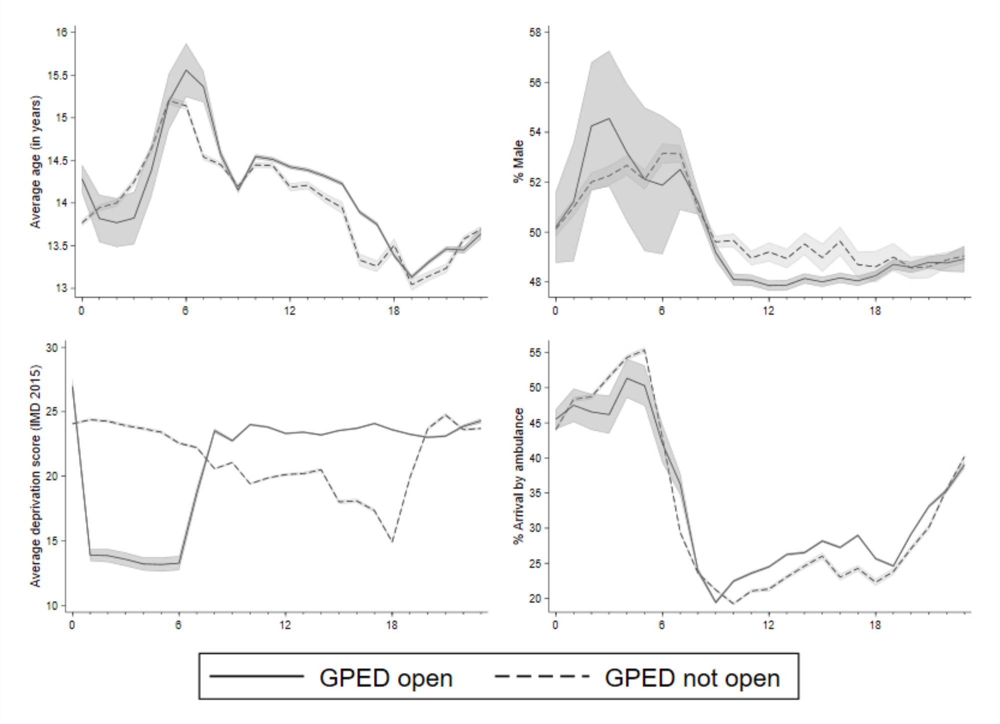

294x213mm (72 x 72 DPI)

STROBE Statement—Checklist of items that should be included in reports of ***cross-sectional studies***

	Item No	Recommendation	Page No
**Title and abstract**	1	(*a*) Indicate the study's design with a commonly used term in the title or the abstract	1
		(*b*) Provide in the abstract an informative and balanced summary of what was done and what was found	1
**Introduction**			
Background/rationale	2	Explain the scientific background and rationale for the investigation being reported	2
Objectives	3	State specific objectives, including any prespecified hypotheses	2
**Methods**			
Study design	4	Present key elements of study design early in the paper	
Setting	5	Describe the setting, locations, and relevant dates, including periods of recruitment, exposure, follow-up, and data collection	2
Participants	6	(*a*) Give the eligibility criteria, and the sources and methods of selection of participants	2-3
Variables	7	Clearly define all outcomes, exposures, predictors, potential confounders, and effect modifiers. Give diagnostic criteria, if applicable	3-5
Data sources/ measurement	8*	For each variable of interest, give sources of data and details of methods of assessment (measurement). Describe comparability of assessment methods if there is more than one group	3-5
Bias	9	Describe any efforts to address potential sources of bias	4-5
Study size	10	Explain how the study size was arrived at	2
Quantitative variables	11	Explain how quantitative variables were handled in the analyses. If applicable, describe which groupings were chosen and why	4-5
Statistical methods	12	(*a*) Describe all statistical methods, including those used to control for confounding	4-5
		(*b*) Describe any methods used to examine subgroups and interactions	5
		(*c*) Explain how missing data were addressed	5
		(*d*) If applicable, describe analytical methods taking account of sampling strategy	n/a
		(*e*) Describe any sensitivity analyses	5
**Results**			
Participants	13*	(a) Report numbers of individuals at each stage of study—eg numbers potentially eligible, examined for eligibility, confirmed eligible, included in the study, completing follow-up, and analysed	3-4
		(b) Give reasons for non-participation at each stage	5
		(c) Consider use of a flow diagram	n/a
Descriptive data	14*	(a) Give characteristics of study participants (eg demographic, clinical, social) and information on exposures and potential confounders	6-8
		(b) Indicate number of participants with missing data for each variable of interest	3-4
Outcome data	15*	Report numbers of outcome events or summary measures	3-4
Main results	16	(*a*) Give unadjusted estimates and, if applicable, confounder-adjusted estimates and their precision (eg, 95% confidence interval). Make clear which confounders were adjusted for and why they were included	8-10

For peer review only - http://bmjopen.bmj.com/site/about/guidelines.xhtml

		(*b*) Report category boundaries when continuous variables were categorized	n/a
		(*c*) If relevant, consider translating estimates of relative risk into absolute risk for a meaningful time period	n/a
Other analyses	17	Report other analyses done—eg analyses of subgroups and interactions, and sensitivity analyses	8-10
**Discussion**			
Key results	18	Summarise key results with reference to study objectives	11
Limitations	19	Discuss limitations of the study, taking into account sources of potential bias or imprecision. Discuss both direction and magnitude of any potential bias	11
Interpretation	20	Give a cautious overall interpretation of results considering objectives, limitations, multiplicity of analyses, results from similar studies, and other relevant evidence	11
Generalisability	21	Discuss the generalisability (external validity) of the study results	11
**Other information**			
Funding	22	Give the source of funding and the role of the funders for the present study and, if applicable, for the original study on which the present article is based	1

*Give information separately for exposed and unexposed groups.

Note: An Explanation and Elaboration article discusses each checklist item and gives methodological background and published examples of transparent reporting. The STROBE checklist is best used in conjunction with this article (freely available on the Web sites of PLoS Medicine at http://www.plosmedicine.org/, Annals of Internal Medicine at http://www.annals.org/, and Epidemiology at http://www.epidem.com/). Information on the STROBE Initiative is available at www.strobe-statement.org.

For peer review only

For peer review only - http://bmjopen.bmj.com/site/about/guidelines.xhtml

BMJ Open

Does the presence of General Practitioners in Emergency Departments affect quality and safety in English NHS Hospitals? A retrospective observational study

Journal:	*BMJ Open*
Manuscript ID	bmjopen-2021-055976.R1
Article Type:	Original research
Date Submitted by the Author:	12-Jan-2022
Complete List of Authors:	Gaughan, James; University of York Centre for Health Economics,<br>Liu, Dan; University of York, Centre for Health Economics<br>Gutacker, Nils; University of York, Centre for Health Economics<br>Bloor, Karen; University of York, Department of Health Sciences<br>Doran, Tim; University of York, Department of Health Sciences<br>Benger, Jonathan; University of the West of England, Faculty of Health and Applied Sciences
**Primary Subject Heading**:	Emergency medicine
Secondary Subject Heading:	General practice / Family practice
Keywords:	Health policy < HEALTH SERVICES ADMINISTRATION & MANAGEMENT, ACCIDENT & EMERGENCY MEDICINE, PRIMARY CARE, HEALTH ECONOMICS

SCHOLARONE™
Manuscripts

I, the Submitting Author has the right to grant and does grant on behalf of all authors of the Work (as defined in the below author licence), an exclusive licence and/or a non-exclusive licence for contributions from authors who are: i) UK Crown employees; ii) where BMJ has agreed a CC-BY licence shall apply, and/or iii) in accordance with the terms applicable for US Federal Government officers or employees acting as part of their official duties; on a worldwide, perpetual, irrevocable, royalty-free basis to BMJ Publishing Group Ltd ("BMJ") its licensees and where the relevant Journal is co-owned by BMJ to the co-owners of the Journal, to publish the Work in this journal and any other BMJ products and to exploit all rights, as set out in our licence.

The Submitting Author accepts and understands that any supply made under these terms is made by BMJ to the Submitting Author unless you are acting as an employee on behalf of your employer or a postgraduate student of an affiliated institution which is paying any applicable article publishing charge ("APC") for Open Access articles. Where the Submitting Author wishes to make the Work available on an Open Access basis (and intends to pay the relevant APC), the terms of reuse of such Open Access shall be governed by a Creative Commons licence – details of these licences and which Creative Commons licence will apply to this Work are set out in our licence referred to above.

Other than as permitted in any relevant BMJ Author's Self Archiving Policies, I confirm this Work has not been accepted for publication elsewhere, is not being considered for publication elsewhere and does not duplicate material already published. I confirm all authors consent to publication of this Work and authorise the granting of this licence.

For peer review only

Does the presence of General Practitioners in Emergency Departments affect quality and safety in English NHS Hospitals? A retrospective observational study

James Gaughan[1], Dan Liu[1], Nils Gutacker[1], Karen Bloor[2], Tim Doran[2], Jonathan Benger[3]

[1] Centre for Health Economics, University of York, York, UK
[2] Health Sciences, University of York, York, UK
[3] Faculty of Health and Applied Sciences, University of the West of England, Bristol, UK

Corresponding Author: James Gaughan, Alcuin 'A' Block, University of York, Heslington, York, UK, YO10 5DD, telephone: 01904 321423, e-mail: james.gaughan@york.ac.uk

Key words: Health policy; Accident and Emergency Medicine; Primary Care; Health Economics

Word count: 3009

For peer review only

Abstract

Objectives
Emergency departments (EDs) in NHS hospitals in England have faced considerable increases in demand over recent years. Most hospitals have developed General Practitioner services in EDs (GPEDs) to treat non-emergency patients, aiming to relieve pressure on other staff and to improve ED efficiency and patient experience. We measured the impact of GPED services on patient flows, health outcomes and ED workload.

Design
Retrospective observational study. Differences in GPED service availability across EDs and time-of-day were used to identify the causal effect of GPED, as patients attending the ED at the same hour of the day are quasi-randomly assigned to treatment or control groups based on their local ED's service availability.

Participants
Attendances to 40 EDs in English NHS hospitals from April 2018 to March 2019, 4,441,349 observations.

Primary and secondary outcomes measured
Outcomes measured were volume of attendances, 'non-urgent' attendances, waiting times over four hours, patients leaving without being treated, unplanned re-attendances within seven days, inpatient admissions and 30-day mortality.

Results
We found a small, statistically significant reduction in unplanned re-attendances within seven days (OR: 0.968, 95% CI: 0.948 to 0.989), equivalent to 302 fewer re-attendances per year for the average ED. The clinical impact of this was judged to be negligible. There was no detectable impact on any other outcome measure.

Conclusions
We found no adverse effects on patient outcomes, neither did we find any evidence of the hypothesised benefits of placing GPs in emergency settings beyond a marginal reduction in re-attendances that was not considered clinically significant.

Strengths and limitations of this study

- Large sample size (all ED attendances at 40 English hospitals over one year)
- Time of patient arrival used to support a causal interpretation
- Wide range of performance measures analysed
- Inability to identify the healthcare professional seen by patients prevented direct comparison
- Data analysis is based on a number of assumptions

Funding: This study was funded by the National Institute of Health Research (NIHR) Health Services and Delivery Research (HS&DR) Programme, project numbers 15/145/04 and 15/145/06.

Competing Interests: The authors have no competing interests to declare.

Contributorship statement: The study was conceived and designed by JB, KB, TD and NG. JG and DL prepared the dataset. JG and NG analysed the data, with additional interpretation by all other authors. JG drafted the manuscript, which was critically reviewed and revised by all other authors. All authors approved the final submission.

Data Sharing Statement: The deidentified patient-level data used in this study, including information on mortality, were released by the data holders (NHS Digital, Office for National Statistics) under specific data sharing agreements and only for the purpose of this study. The data sharing agreements do not permit further sharing or publication of the data. Interested parties may seek to obtain data directly from the relevant data holders. HES data are copyright 2018-2019, re-used with the permission of NHS Digital through Data Sharing Agreement NIC-84254-J2G1Q. Data collected about the hours a GPED service was collected by the authors specifically for this project and are not able to place the original data into the public domain.

Ethics Statement: This research study was approved by:

- East Midlands - Leicester South Research Ethics Committee (Ref: 17/EM/0312)
- University of Newcastle Ethics Committee (Ref: 14348/2016)
- Health Research Authority (HRA) (IRAS: 230848 and 218038).

For peer review only

Introduction

Emergency department crowding has been described as a 'worldwide public health problem'[1] and even an 'international crisis'.[2] Crowding, which has been linked with various measures of quality of care,[3] can result from the volume of patients attending (input), delays in the emergency department (throughput) or blocks to patients leaving (output)[4] - for example, problems with accessing hospital beds or social care support.

In common with many health care systems, emergency departments (EDs) in the English NHS have faced considerable growth in demand over recent years.[5] Efforts to reduce unnecessary attendances to EDs (input), such as the introduction of walk-in primary care centres and NHS telephone and online advice services, have been shown to address unmet needs but have not changed trends in ED attendance.[6] Policy attention has consequently switched to throughput of patients inside the ED. A substantial minority of attendances are suitable for treatment by a primary care physician – known in the UK as a General Practitioner (GP); Mason et al[7] estimated this proportion to be around 23% of adults and 31% of children attending EDs in England. Following the recommendations of a national review of emergency services,[8] most acute NHS hospitals have developed GP services in EDs (GPEDs) to treat non-urgent patients, aiming to relieve pressure on other ED staff and improve ED efficiency and patient experience.[9 10] Different models of GPs working in or alongside the ED have emerged; some are fully integrated within the ED, others 'stream' patients either to GPs working alongside EDs or to outside services, which may be offered on site or off site.[11]

Evidence of the effectiveness of GP or other primary care practitioners in EDs is limited to date. A 2018 Cochrane review found only four studies (one randomised trial and three non-randomised studies) that evaluated the effects of introducing GPs or emergency nurse practitioners.[12] These studies did not examine safety and had inconsistent results, leading reviewers to comment that the evidence was insufficient to draw conclusions for practice or policy. The Cochrane review has relatively stringent methodological inclusion criteria (randomised trials, interrupted time series and controlled before and after studies only), but more inclusive reviews have similarly found inadequate evidence on the effectiveness of this approach.[13 14] A narrative review including all study types from seven countries (Netherlands, England, Australia, Ireland, Spain, Sweden and Switzerland) [13] described a paradoxical increase in attendances, which the authors attributed to provider-induced demand, and they concluded that any marginal savings per patient are likely to be overshadowed by the overall cost of the new service. A realist review including studies from the UK, six EU countries, Australia, USA, Canada, Singapore and New Zealand[14] found that GPEDs may shorten process times for non-urgent patients, but that there is little evidence that this frees up emergency department staff time; this study also raised potential concerns about provider-induced demand.

As part of a mixed methods study to explore the impacts of General Practitioners working in or alongside the emergency department,[15] we analysed data from hospitals in England with a well-established and clearly defined GPED service during the financial year April 2018 to March 2019, using the presence of a GP service as the intervention. The study aim was to measure the impact of GPED services on patient throughput in the ED (including waiting time, treatment in the ED and unplanned re-attendance). Given the association between ED crowding and the quality and safety of patient care,[3] we also investigated patient health outcomes, including 30-day mortality.

For peer review only

Methods

Study design and data sources

We undertook a retrospective observational study of all clinical activity in Type 1 emergency departments (i.e. EDs that are open 24 hours a day and led by a Consultant in Emergency Medicine) in English NHS hospital trusts during the period 01 April 2018 to 31 March 2019. Complete data on availability and type of GPED service, derived from a national mapping survey,[16] were available for 40 of England's 137 Type 1 emergency departments. The unit of analysis was an individual ED attendance.

The primary data source was the Hospital Episode Statistics Accident & Emergency (HES A&E) dataset.[17] HES A&E is an administrative dataset which reports information about all attendances to all hospital emergency departments in England and forms the basis for hospital reimbursement. The dataset provides information on patients' socio-demographic characteristics (age (in years), sex, and local area deprivation profile based on the Index of Multiple Deprivation 2019[18]). There were 4,610,364 attendances to the 40 EDs for which we observed GPED availability times. Of these, we excluded 116,316 (2.5%) observations with missing deprivation score due to unknown patient place of residence; 333 (0.01%) observations with missing sex; and 52,366 (1.14%) observations with missing age. This left a main sample of 4,441,349 observations. The timing of patient attendance and discharge from ED (recorded as date, hour and minute) and the discharge destination are also included within the dataset. Full technical details of variables within the HES dataset can be found at (https://digital.nhs.uk/data-and-information/data-tools-and-services/data-services/hospital-episode-statistics/hospital-episode-statistics-data-dictionary).

Information on the date of death for deceased patients was provided by the Office for National Statistics and linked to HES A&E by NHS Digital.[19]

HES A&E data do not identify the treating healthcare professional and it was therefore not possible to ascertain whether a patient was seen by a GP or another member of staff. Hospital-reported GPED opening hours were used instead to assign ED attendances to treatment and control groups based on the potential exposure to GPED services at the patient's time of arrival to the ED. Data on opening and closing hours of GPED services for individual hospitals by day of the week were obtained via the mapping survey.[16] EDs were also asked to identify their local model of GPED service, which was pre-specified to be either: i) integrated, with the GP working as part of the ED team; ii) parallel, where the GP service is separate from but located adjacent to the ED; or iii) off site, with the GP service provided at a separate site on the same hospital campus. Only hospital EDs that provided complete data as part of this national survey were included in the analyses.

Outcome measures

We investigated the impact of GPED on a range of different measures of ED performance and patient outcomes (*Table 1*). All outcome measures were defined at individual patient level except volume of activity, which is measured at ED-hour-day level. Outcomes could not be calculated for all attendances due to missing information recorded in the HES dataset. For example, discharge destination was not recorded for 14% of patients so that outcomes 3 ('Untreated') and 5 ('Admitted to ward') could not be calculated for these patients. For outcomes 4 ('Non-urgent attendance') and 6 ('30-day mortality') missingness could not be ascertained because Information about the outcome is defined as the presence of some recorded information. If this information is absent, it was assumed that the outcome had not occurred. Finally, attendances occurring within the final 7 (30) days of the study period were not included when analysing unplanned reattendances (30-day mortality) as re-attendance or death might have occurred within the relevant time-frame but outside the study period.

Information about the specific HES A&E variables, the values used to identify each outcome, and the reasons for missingness in each outcome measure, are presented in Appendix A. Observations with missing outcome information were only excluded from the statistical analysis of the relevant outcome.

For peer review only

Table 1: Outcome measures and summary statistics for all EDs in England and those included in the analysis.

ID	Outcome	Definition	Unit of analysis	Included EDs (n=40)		Excluded EDs (n=97)		Missing (%) (included EDs only)
				Proportion of patients (%)	Observations (millions)	Proportion of patients (%)	Observations (millions)	
Patient flow and outcomes								
1	Wait over 4 hours	Time from admission to discharge was more than four hours.	Patient	16.49	4.4	19.77	11.6	0.6
2	Unplanned re-attendance	Patient made an unplanned re-attendance to the same or different ED within 7 days of discharge.	Patient	9.01	4.2	8.67	11.1	3.5
3	Untreated	Patient left ED without being treated.	Patient	2.36	3.8	2.13	10.8	14.2
4	Non-urgent attendance[7] [20]	An attendance is 'unnecessary' or 'non-urgent' if **all** of the following conditions are met:<br>• No Investigations were recorded or they were limited to urinalysis, pregnancy test, or dental investigation.<br>And	Patient	11.50	4.4	10.69	11.6	unknown

For peer review only

		• No treatments were recorded or were limited to guidance, written advice, verbal advice, recording of vital signs, dental treatment, or prescription of medicines.<br>And						
		• Treatment or follow-up was limited to primary care or patient left before being treated.<br>And						
		• Attendance was not by ambulance.						
5	30-day mortality	Date of death of the patient is within 30 days after ED attendance.	Patient	1.58	4.1	1.74	10.6	unknown
6	Admission to ward	Patient admitted as an inpatient following ED attendance.	Patient	23.65	3.8	23.52	10.8	14.2
ED workload								
ID	Outcome	Definition	Unit of analysis	Included EDs (n=40)		Excluded EDs (n=97)		Missing (%) (included EDs only)
				Mean (SD)	Observations (000's)	Mean (SD)	Observations (000's)	
7	Volume of attendances	Count of attendances per hour of day and day of week	Provider-hour-day	12.68 (9.13)	350	13.37 (11.59)	850	0.0

For peer review only

Statistical analysis

The statistical analysis relies on differences in GPED service availability across EDs and time-of-day to identify the causal effect of GPED on outcomes. Hospitals operate different opening hours for their GPED services and few offer 24/7 coverage. For example, a service might begin at 8am and end at 11pm in one hospital but run from 12pm to 8pm in another hospital. As such, at a given hour, patients attending some EDs will have access to GPED services whereas patients attending some other EDs will not. As patients are likely to attend the ED nearest to their usual place of residence and are unlikely to plan the timing of their attendance, this created a natural experiment in which patients attending the ED at the same hour of the day are quasi-randomly assigned to treatment or control groups based on their local GPED service availability. To estimate the impact of GPED availability, we consider all 24 hours of the day over the full study period from 01 April 2018 to 31 March 2019.

Logit and Poisson regression models were estimated to identify the effect of treatment status on outcomes at patient and provider level. Each outcome was analysed separately. All models controlled for hospital and hour-by-day of week fixed effects, month of year fixed effects, and, in the case of the attendance-level regressions, potential residual confounders in the form of patient age (coded as 0, 1, 2 ,3, 4, 5, 6-10, 11-15 … 91-95, 96+), sex, age-sex interactions, an indicator for arrival by ambulance, and area-level socio-economic deprivation based on place of residence (Index of Multiple Deprivation (IMD) score grouped in equal quintiles. The hospital fixed effects capture time-invariant differences in performance across EDs that reflect hospital-specific factors such as management quality, building infrastructure, and the quality and availability of substitute healthcare services within the local health economy.[21] The hour-by-day fixed effects capture differences in service availability and patient acuity over the course of the day that follow a common pattern across all hospitals in England. Attendances in the last 7 or 30 days of the sample period were excluded from analysis for outcomes 7-day re-attendance and 30-day mortality, respectively.

Our main analyses assumed a common effect across GPED models. Secondary analyses allowed for subgroup effects by type of GPED model.

The estimation of the impact of GPED was limited to time periods where there was variation in GPED service availability across hospitals. While all attendances to analysed EDs in the study period were analysed, patients attending EDs during hours of the day when all/none of the EDs operated a GPED service contributed to the identification of hospital fixed effects and the effect of observed confounders but did not contribute to the statistical identification of the effect of GPED services on outcomes. This group includes 257,532 attendances (5.8% of the full sample.

Estimates are reported as odds ratios (ORs) or incidence rate ratios (IRRs) with associated 95% confidence intervals (CIs). Figures are rounded to the third decimal due to the large number of ED attendances. Bonferroni-Dunn correction was applied to adjust CIs for multiple testing while ensuring an overall family-wise error rate of 5%.[22] Standard errors are clustered at ED level. All analyses were performed in Stata version 16 (StataCorp, College Station, TX, USA).

Patient and public involvement

We held meetings with a patient and public Research Advisory Group throughout the study. We discussed the study methods with this group and presented results for feedback at various stages, modifying the research and its interpretation in response to their feedback. Our final results were disseminated to the Research Advisory Group and others through a collaborative workshop. Our

For peer review only

study was an analysis of secondary data; therefore, we did not recruit patients as research participants.

Results

GPED services

Data on GPED service opening hours and GPED model were available for 40 EDs, with a total of 4.6m attendances between April 2018 and March 2019. Most EDs had a GP present between 10am and 10pm on all days of the week (Figure 1), which coincides with the time of day in which workload tends to be highest (Figure 2). Some hospitals operate different GPED service opening hours during weekdays and weekends, with two hospitals offering no GPED services on weekends. One or more GPs were present in EDs in our sample for a median of 13 hours (IQR: 11 to 15) on weekdays and 13 hours (IQR: 9 to 15) on weekends.

Demographic characteristics of patients attending EDs where a GPED service was available and where GPED was not available were similar, on average, and followed a common pattern over the course of the day (*Figure 3*). The only exception is deprivation, with patients attending EDs where a GPED service was available during the early hours of the day being, on average, from less deprived neighbourhoods.

For peer review only

Figure 1 about here

Figure 2 about here

Figure 3 about here

Impact of GPED

The results of the main analysis are presented in table 2. There was no statistically significant effect of GPED on any of the outcome measures aside from a small, statistically significant reduction in unplanned re-attendances within seven days (OR: 0.968, 95% CI (unadjusted): 0.948 to 0.989). This effect was only detectable for integrated and parallel GPED services, and not for off-site GP services. None of these findings reach statistical significance when adjusted for multiple testing.

Table 2: Impact of GPED service availability on outcomes, overall and by GPED model

**Outcome / GPED model**	**OR / IRR**	**95% CI (unadjusted)**	**95% CI (adjusted for multiple testing)**
Wait over 4 hours (N = 4,417,155 attendances)			
Overall	1.012	0.927 to 1.105	0.870 to 1.153
Integrated	0.906	0.793 to 1.034	0.694 to 1.118
Parallel	0.997	0.863 to 1.132	0.783 to 1.211
Off-site	1.077	0.972 to 1.182	0.910 to 1.244
Unplanned re-attendance within 7 days (N = 4,198,299 attendances)			
Overall	0.968	0.948 to 0.989	0.935 to 1.001
Integrated	0.930	0.891 to 0.973	0.860 to 1.000
Parallel	0.972	0.945 to 0.998	0.929 to 1.014
Off-site	0.980	0.950 to 1.009	0.933 to 1.027

For peer review only

Untreated (N = 3,793,246 attendances)).			
Overall	0.913	0.787 to 1.060	0.696 to 1.130
Integrated	0.976	0.837 to 1.142	0.728 to 1.224
Parallel	0.926	0.730 to 1.124	0.611 to 1.241
Off-site	0.787	0.620 to 0.955	0.520 to 1.055
Non-urgent attendance (N = 4,441,349 attendances)			
Overall	1.038	0.944 to 1.142	0.881 to 1.196
Integrated	1.014	0.844 to 1.219	0.722 to 1.306
Parallel	1.092	0.999 to 1.187	0.942 to 1.242
Off-site	0.990	0.874 to 1.106	0.805 to 1.175
Admission to ward (N = 3,793,246 attendances)			
Overall	1.029	0.957 to 1.106	0.910 to 1.148
Integrated	1.039	1.016 to 1.185	0.916 to 1.161
Parallel	1.002	0.886 to 1.106	0.807 to 1.198
Off-site	1.047	0.957 tot 1.171	0.895 to 1.199
30-day mortality (N = 4,076,605 attendances)			
Overall	1.014	0.990 to 1.038	0.975 to 1.052
Integrated	1.041	0.968 to 1.119	0.926 to 1.156
Parallel	1.017	0.972 to 1.061	0.947 to 1.087

For peer review only

Off-site	1.000	0.968 to 1.032	0.948 to 1.053
Volume of attendances (N = 342,940 hospital-hour-day combinations)).			
Overall	1.000	0.967 to 1.034	0.946 to 1.054
Integrated	0.983	0.914 to 1.053	0.873 to 1.094
Parallel	1.018	0.967 to 1.069	0.937 to 1.099
Off-site	0.986	0.938 to 1.034	0.910 to 1.062

Notes: All models controlled for hospital and hour-by-day of week fixed effects, month of year fixed effects, and, in the case of the attendance-level regressions, patient age (coded as 0, 1, 2 ,3, 4, 5, 6-10, 11-15 … 91-95, 96+), sex, age-sex interactions, an indicator for arrival by ambulance, and socio-economic deprivation (IMD) quintiles. Observations are dropped if any of the dependant or independent variables are missing. See Appendix A for details.

Discussion

Statement of principal findings

Many hospitals in England have implemented GPED services with the aim of treating non-urgent patients more appropriately, thereby improving efficiency and patient experience. Using attendance data for 2018/19, we found no statistically significant effect of GPED services on volume of attendances or on most performance indicators, including four-hour waits, patients leaving without being treated, or hospital admissions. We did identify a small, statistically significant reduction in unplanned re-attendances within seven days, equivalent to 302 fewer re-attendances per year for the average ED. This is less than one prevented re-attendance per day for the average ED. The finding was not statistically significant after adjusting for multiple testing, and was judged to be of negligible clinical significance.

Strengths and weaknesses

Our analysis exploited differences in the implementation of GPED services across 40 hospitals in terms of time of operation to model robustly their impact on a range of key indicators, accounting for a wide range of covariates. Using information from targeted surveys, we were also able to perform a sub-group analysis for three different GPED models, strengthening the evidence base to inform decisions about implementing GPED services. The use of time of day on which to base our analysis avoids a number of potential confounding factors, such as general hospital crowding or lack of community support.

Our analysis was, however, constrained in a number of ways. Most importantly, it was not possible to identify from available data which staff members assessed and treated individual patients, so we could not separate patients treated by GPs from those treated by other ED staff to directly compare GP services to traditional models of care. We relied primarily on measures of general ED performance, such as attendances, patient flow and waiting times. Our approach assumed GPs were present during the working hours reported in the survey, but there is potential for misclassifying

For peer review only

patient episodes as GPED/not GPED as streaming activity and GP availability may not have corresponded exactly to official GPED start and finish times, particularly for patients arriving close to these times or undergoing extended waits. The start and finish times of GPED shifts may also have coincided with the shifts of other health professionals, such as advanced clinical practitioners, which have also been introduced to some EDs. Our design relies upon no other major differences in service provision occurring at the same time as GPED shift hours. Our analysis also assumes that GPED was always operational during designated hours and therefore does not take account of physician absence. There were differences in observed outcomes between hospitals included in our analysis and those excluded from analysis (Table 1). There were also substantial differences between the socioeconomic deprivation profile of patients attending hospitals with and without GPED services in the early hours of the day (Figure 3). Finally, we were not able to assess the degree of 'role substitution', i.e. whether GPs were an addition to normal services, or whether they replaced another member of staff.

Principal findings in context of other studies

We found that patients attending emergency departments during the hours of operation of a GPED service had similar outcomes to patients attending at other times, with the possible exception of a very small reduction in unplanned re-attendances within seven days. Previous evidence on the impact of such services is extremely limited, and no previous studies have examined interventions on the same scale. A 2018 Cochrane review found no clear evidence that deploying primary care physicians in emergency departments reduced treatment times, lengths of ED stay, safety, hospital admissions, resource use, cost-effectiveness or referrals.

Future research in this area could usefully explore the implementation of GPED services, using more observational and qualitative methods, and include other important outcome measures (for example, those reflecting patient experience). which could improve data collection in EDs The cost implications of these services should also be investigated further. Employing GPs in EDs has opportunity costs in terms of both alternative use of funds for urgent and emergency care (for example, employing additional nurses, physician associates or emergency physicians) and loss of GPs from primary care settings. Prior to the COVID-19 pandemic, there was a trend of increasing demand for both ED and primary care services, and general practice is an area of potential workforce shortages, with a forecasted shortfall of 7,000 GPs in the next five years.[23] This raises further questions about the appropriateness of recruiting GPs to work in hospital ED settings.

Conclusions

We found no adverse effects on patient outcomes, neither did we find any evidence of the hypothesised benefits of placing GPs in emergency settings beyond a marginal reduction in re-attendances that was not considered clinically significant. Given that GPED services are likely to incur significant additional costs, and to take GPs from primary care services which are themselves under pressure, we found no evidence that they are a good use of scarce health care resources in the absence of further development of the model and substantial improvements in outcomes.

For peer review only

References

1. Pines J, Griffey R. What we have learned from a decade of ED crowding research? *Acad Emerg Med* 2015;22(8):7.
2. Hoot N, Aronsky D. Systematic review of emergency department crowding: Causes effects and solutions. *Ann Emerg Med* 2008;52(2):10.
3. Stang A, Crotts J, Johnson D, et al. Crowding measures associated with the quality of emergency department care: A systematic review. *Acad Emerg Med* 2015;22:14.
4. Morley C, Unwin M, Peterson G, et al. Emergency department crowding: A systematic review of causes consequences and solutions. *PLOS One* 2018;13(8)
5. National Audit Office. Emergency admissions to hospital: managing the demand, 2013.
6. Turner J, O'Cathain A, Knowles E, et al. Impact of the urgent care telephone service NHS 111 pilot sites: a controlled before and after study. *BMJ Open* 2013;3(11):e003451. doi: 10.1136/bmjopen-2013-003451
7. Mason S, O'Keeffe C, Jacques R, et al. Perspectives on the reasons for Emergency Department attendnaces acrss Yorkshire and the Humber - Final Report September 2017. The University of Sheffield, 2017.
8. NHS England. High quality care for all, now and for future generations: Transforming urgent and emergency care services in England - Urgent and Emergency Care Review End of Phase 1 Report. Leeds, 2013.
9. NHS England. Next steps on the NHS Five Year Forward View, 2017.
10. NHS England. Transforming urgent and emergency care services in England - Safer, faster, better: good practice in delivering urgent and emergency care. London, 2016.
11. Cooper A, Edwards M, Brandling J, et al. Taxonomy of the form and function of primary care services in or alongside emergency departments: concepts paper. *Emergency Medicine Journal* 2019;36(10):625-30. doi: 10.1136/emermed-2018-208305
12. Gonçalves-Bradley D, Khangura JK, Flodgren G, et al. Primary care professionals providing non-urgent care in hospital emergency departments. *Cochrane Database Syst Rev* 2018;2(2):Cd002097. doi: 10.1002/14651858.CD002097.pub4
13. Ramlakhan S, Mason S, O'Keeffe C, et al. Primary care services located with EDs: a review of effectiveness. *Emergency Medicine Journal* 2016;33(7):495-503. doi: 10.1136/emermed-2015-204900
14. Cooper A, Davies F, Edwards M, et al. The impact of general practitioners working in or alongside emergency departments: a rapid realist review. *BMJ Open* 2019;9(4):e024501. doi: 10.1136/bmjopen-2018-024501
15. Morton K, Voss S, Adamson J, et al. General practitioners and emergency departments (GPED) - efficient models of care: A mixed-methods study protocol. *BMJ Open* 2018;8:8. doi: 10.1136/bmjopen-2018-024012
[dataset] 16. Brant H, Voss S, Morton K, et al. Current provision of general practitioner services in or alongside emergency departments in England. *Emergency Medicine Journal* 2021;38:4. doi: 10.1136/emermed-2020-210539
[dataset] 17. NHS Digital. Hospital Episode Statistics (HES) 2022 [updated 05.01.2022. Available from: https://digital.nhs.uk/data-and-information/data-tools-and-services/data-services/hospital-episode-statistics accessed 10.01.2022 2022.
18. Noble S, McLennan D, Noble M, et al. The English Indices of Deprivation 2019. In: Ministry of Housing CLG, ed. London, 2019.
[dataset] 19. NHS Digital. Linked HES-ONS mortality data 2020 [updated 15.01.2020. Available from: https://digital.nhs.uk/data-and-information/data-tools-and-services/data-services/linked-hes-ons-mortality-data accessed 10.01.2022 2022.
20. NHS Digital. Non-urgent A&E attendances 2021 [Available from: https://digital.nhs.uk/data-and-information/data-tools-and-services/data-services/innovative-uses-of-data/demand-on-healthcare/unnecessary-a-and-e-attendances accessed 16/11/2021.
21. Wooldridge JM. Econometric Analysis of Cross Section and Panel Data. Cambridge, Massachusetts: MIT Press 2002.
22. Dunn OJ. Multiple Comparisons among Means. *Journal of the American Statistical Association* 1961;56(293):52-64. doi: 10.1080/01621459.1961.10482090
23. Beech J, Bottery S, Charlesworth A, et al. Closing the gap - Key areas for action on the health and care workforce. London: The King's Fund, 2019.

For peer review only

Figure Legend

Fig 1: GPED service availability by hour of day
Fig 2: Volume of attendances by hour of day
Fig 3: Patient characteristics by hour of day (mean, 95% CI)

For peer review only

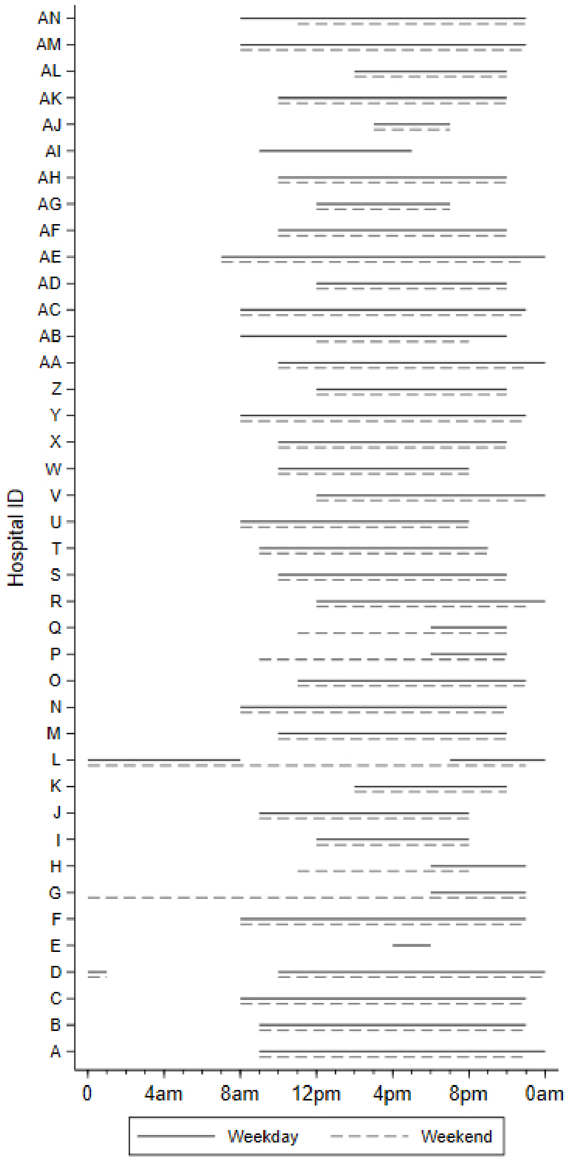

Fig 1: GPED service availability by hour of day

136x273mm (72 x 72 DPI)

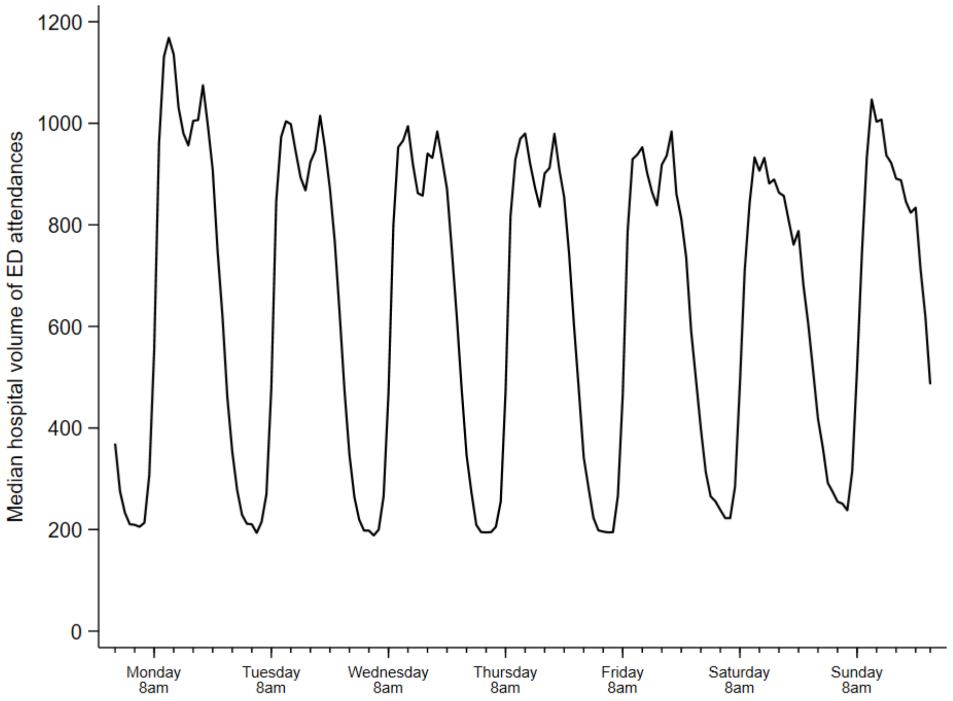

Fig 2: Volume of attendances by hour of day

342x257mm (72 x 72 DPI)

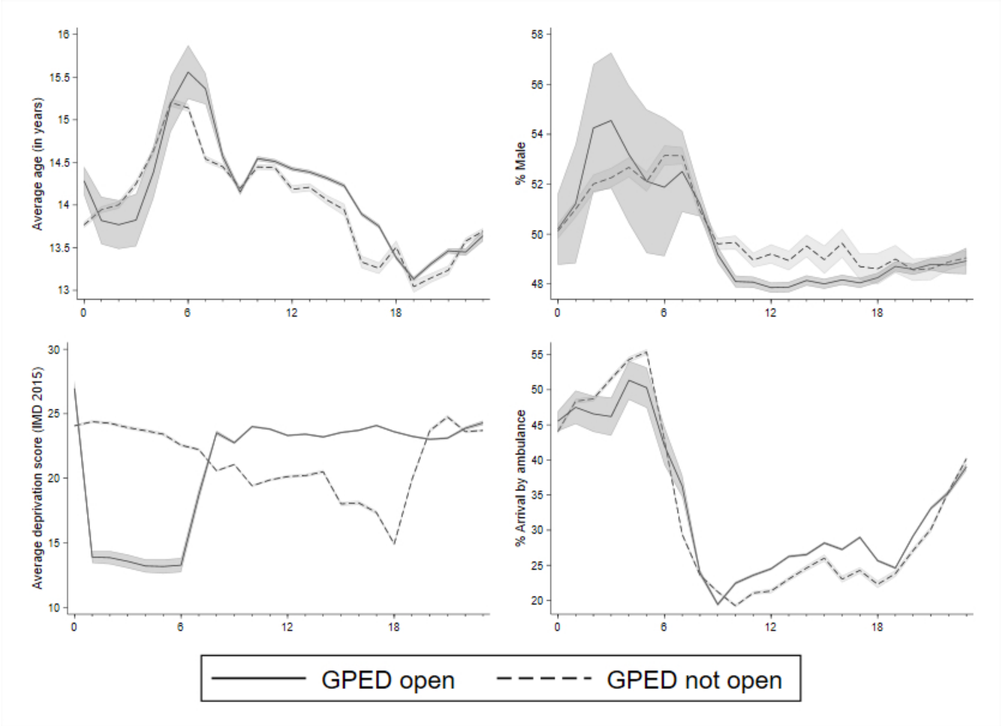

Fig 3: Patient characteristics by hour of day (mean, 95% CI)

294x213mm (72 x 72 DPI)

Appendix A: Detailed description of outcome definitions

ID	Outcome name	Algorithm (variables used in parentheses)	Causes of missing values
1	Wait over 4 hours	= 1 if the time from admission to discharge (depdur) in an ED is greater than four hours.	depdur is missing (value of .)
2	Unplanned re-attendance within 7 days	= 1 if the patient (extract_hesid) re-attended an ED within 7 days of discharge (difference in arrivaldate) and that reattendance was not planned (aeattendcat = 1 or 3).	aeattendcat is unknown (value = 9)
3	Untreated	= 1 if the attendance is unplanned (aeattendcat =1 or 3) and the patient leaves before being treated (aeattenddisp = 12)	Aeattendcat is unknown (value 9) or aeattenddisp is unknown (value 99)
4	'Unnecessary' attendance	=1 if all of the following conditions are met:<br><br>1. Any investigations (invest_nn) reported are for one of (have the first two characters) "urin analysis" 06, "pregnancy test" "21", dental investigation" (22) "none" (24).<br><br>2. Any treatments (treat_nn) are limited to (a recorded values of) "guidance/advice only-written" (221), "guidance/advice only-verbal (222), "recording vital signs" (30), "dental treatment" (56), "prescription/medicine prepared to take away (57), "none" (99)<br><br>3. Patient not treated or any follow-up limited to primary care (aeattenddisp values of) "discharged-follow up	n/a, as it is not possible to disentangle nul from missing values.

		treatment to be provided by general practitioner (02), "discharged did not require any follow-up treatment" (03), "left department before being treated" (12)<br><br>4. Attendance is not by ambulance (aearrivalmodel doesn't equal 1)	
5	Admission to ward	=1 if patient is admitted as an inpatient following ED attendance (aeattenddisp = 1 or 7).	aeattenddisp is unknown (value 99)
6	30-day mortality	=1 if date of death of the patient is within 30 days after ED attendance.	
7	Volume of attendances	Count of attendances per hour of day and day of week	

For peer review only

STROBE Statement—Checklist of items that should be included in reports of ***cross-sectional studies***

	Item No	Recommendation	Page No
**Title and abstract**	1	(*a*) Indicate the study's design with a commonly used term in the title or the abstract	1
		(*b*) Provide in the abstract an informative and balanced summary of what was done and what was found	1
**Introduction**			
Background/rationale	2	Explain the scientific background and rationale for the investigation being reported	2
Objectives	3	State specific objectives, including any prespecified hypotheses	2
**Methods**			
Study design	4	Present key elements of study design early in the paper	
Setting	5	Describe the setting, locations, and relevant dates, including periods of recruitment, exposure, follow-up, and data collection	2
Participants	6	(*a*) Give the eligibility criteria, and the sources and methods of selection of participants	2-3
Variables	7	Clearly define all outcomes, exposures, predictors, potential confounders, and effect modifiers. Give diagnostic criteria, if applicable	3-5
Data sources/ measurement	8*	For each variable of interest, give sources of data and details of methods of assessment (measurement). Describe comparability of assessment methods if there is more than one group	3-5
Bias	9	Describe any efforts to address potential sources of bias	4-5
Study size	10	Explain how the study size was arrived at	2
Quantitative variables	11	Explain how quantitative variables were handled in the analyses. If applicable, describe which groupings were chosen and why	4-5
Statistical methods	12	(*a*) Describe all statistical methods, including those used to control for confounding	4-5
		(*b*) Describe any methods used to examine subgroups and interactions	5
		(*c*) Explain how missing data were addressed	5
		(*d*) If applicable, describe analytical methods taking account of sampling strategy	n/a
		(*e*) Describe any sensitivity analyses	5
**Results**			
Participants	13*	(a) Report numbers of individuals at each stage of study—eg numbers potentially eligible, examined for eligibility, confirmed eligible, included in the study, completing follow-up, and analysed	3-4
		(b) Give reasons for non-participation at each stage	5
		(c) Consider use of a flow diagram	n/a
Descriptive data	14*	(a) Give characteristics of study participants (eg demographic, clinical, social) and information on exposures and potential confounders	6-8
		(b) Indicate number of participants with missing data for each variable of interest	3-4
Outcome data	15*	Report numbers of outcome events or summary measures	3-4
Main results	16	(*a*) Give unadjusted estimates and, if applicable, confounder-adjusted estimates and their precision (eg, 95% confidence interval). Make clear which confounders were adjusted for and why they were included	8-10

For peer review only - http://bmjopen.bmj.com/site/about/guidelines.xhtml

		(*b*) Report category boundaries when continuous variables were categorized	n/a
		(*c*) If relevant, consider translating estimates of relative risk into absolute risk for a meaningful time period	n/a
Other analyses	17	Report other analyses done—eg analyses of subgroups and interactions, and sensitivity analyses	8-10
**Discussion**			
Key results	18	Summarise key results with reference to study objectives	11
Limitations	19	Discuss limitations of the study, taking into account sources of potential bias or imprecision. Discuss both direction and magnitude of any potential bias	11
Interpretation	20	Give a cautious overall interpretation of results considering objectives, limitations, multiplicity of analyses, results from similar studies, and other relevant evidence	11
Generalisability	21	Discuss the generalisability (external validity) of the study results	11
**Other information**			
Funding	22	Give the source of funding and the role of the funders for the present study and, if applicable, for the original study on which the present article is based	1

*Give information separately for exposed and unexposed groups.

Note: An Explanation and Elaboration article discusses each checklist item and gives methodological background and published examples of transparent reporting. The STROBE checklist is best used in conjunction with this article (freely available on the Web sites of PLoS Medicine at http://www.plosmedicine.org/, Annals of Internal Medicine at http://www.annals.org/, and Epidemiology at http://www.epidem.com/). Information on the STROBE Initiative is available at www.strobe-statement.org.

For peer review only
